# Examination of gametocyte protein 22 localization and oocyst wall formation in *Eimeria necatrix* using laser confocal microscopy and scanning electron microscopy

**DOI:** 10.1186/s13071-023-05742-z

**Published:** 2023-04-12

**Authors:** Lele Wang, Dandan Liu, Yang Gao, Zhaofeng Hou, Yu Zhu, Feiyan Wang, Wenjing Li, Amin Zhang, Jinjun Xu, Junjie Hu, Jianping Tao

**Affiliations:** 1 College of Veterinary Medicine, Yangzhou University, 12 East Wenhui Road, Yangzhou, 225009 Jiangsu People’s Republic of China; 2 Jiangsu Co-Innovation Center for Prevention and Control of Important Animal Infectious Diseases and Zoonoses, Yangzhou University, Yangzhou, 225009 People’s Republic of China; 3 Joint International Research Laboratory of Agriculture and Agri-Product Safety, The Ministry of Education of China, Yangzhou University, Yangzhou, 225009 People’s Republic of China; 4 School of Ecology and Environmental Sciences and Yunnan Key Laboratory for Plateau Mountain Ecology and Restoration of Degraded Environments, Yunnan University, Kunming, 650091 People’s Republic of China

**Keywords:** Eimeria necatrix, Gametocyte proteins, Oocyst wall formation, Wall-forming bodies

## Abstract

**Background:**

*Eimeria* parasite infection occurs via ingestion of oocysts. The robust, bilayer oocyst wall is formed from the contents of wall-forming bodies (WFBs), WFB1 and WFB2, located exclusively in macrogametocytes. *Eimeria necatrix* gametocyte proteins 22 and 59 (EnGAM22 and EnGAM59) have been found to localize to WFBs and the oocyst wall. However, the exact localization of these two proteins is not clear.

**Methods:**

WFBs of *E. necatrix* were extracted from purified gametocytes using a cutoff filter and the extracts of purified WFBs and gametocytes were analyzed using sodium dodecyl sulfate–polyacrylamide gel electrophoresis (SDS-PAGE) and immunoblotting. Then, the localization of EnGAM22 and EnGAM59 proteins was determined using an indirect immunofluorescence assay. Finally, the development of macrogametocytes and the oocyst wall of *E. necatrix* was analyzed using laser confocal microscopy and scanning electron microscopy.

**Results:**

Purified WFBs had the same shape and size as those observed in macrogametocytes. EnGAM22 protein localized to WFB1, whereas EnGAM59 protein localized to WFB2. Both EnGAM22 and EnGAM59 native proteins were detected in the extracts of WFBs and gametocytes. The outer layer of the oocyst wall was formed by the release of the contents of WFB1 at the surface of the macrogametocyte to form an anti-EnGAM22 positive layer. WFB2 then appeared to give rise to the inner layer, which was anti-EnGAM59 positive.

**Conclusions:**

EnGAM22 and EnGAM59 proteins localized to WFB1 and WFB2 and were involved in the formation of the outer and inner layers of the oocyst wall of *E. necatrix,* respectively. The processes of macrogametogenesis and oocyst wall formation of *E. necatrix* are similar to other *Eimeria* parasites. The anti-EnGAM22 antibody could be used as a tool to track the transport and secretion of proteins in WFB1 during oocyst development.

**Graphical Abstract:**

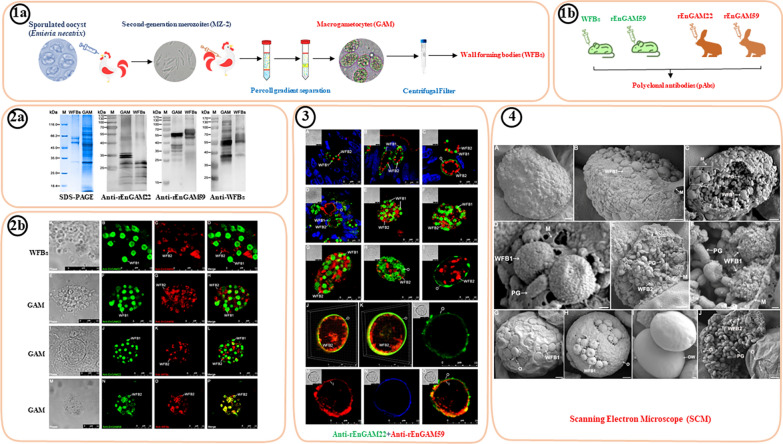

**Supplementary Information:**

The online version contains supplementary material available at 10.1186/s13071-023-05742-z.

## Background

Avian coccidiosis is an important disease caused by one or more of seven species of the genus *Eimeria*, including *Eimeria tenella*, *E. necatrix*, *E. acervulina*, *E. maxima*, *E. brunetti*, *E. praecox*, and *E. mitis*. These have caused significant losses to the chicken industry, including losses during production and costs for prophylaxis and treatment, with an estimated global cost of approximately £10.4 billion at 2016 prices, equivalent to £0.16 per chicken produced [1]. *Eimeria necatrix* is a highly pathogenic coccidium that can cause high rates of mortality in susceptible birds, particularly in chickens older than 8 weeks raised on a litter floor [2].

*Eimeria* infections occur via ingestion of oocysts [3]. Oocysts that develop from macrogametes are encapsulated by a hard barrier, the oocyst wall, which protects the parasite from the harsh external environment [3, 4]. The basic processes of macrogametogenesis and oocyst wall formation have been described in *E. tenella* [5, 6], *E. necatrix* [7, 8], *E. acervulina* [9, 10], *E. maxima* [11–13], and *E. brunetti* [14]. It was shown that two types of wall-forming bodies (WFBs), WFB1 and WFB2, were synthesized during the maturation of the macrogamete, and these appeared to give rise to the outer and inner layers of the oocyst wall, respectively. In addition, a loose veil enclosing the developing intracellular oocyst appeared to be lost during excretion in the feces [3, 4].

The oocyst wall of *Eimeria* is primarily made up of protein (> 90%), but only several proteins have been identified [4]. The best characterized of these are two tyrosine-rich gametocyte proteins (GAM56 and GAM82) that were identified and localized to WFB2 and the inner oocyst wall of *Eimeria maxima* [15–19], *E. tenella* [19, 20], *E. acervulina* [19], and *E. nieschulzi* [21]. In addition, a *gam56 tmp2* (or *gam59*) gene that encodes a second GAM56-like protein (GAM59) exists in the genomes of *E. tenella* [19, 22], *E. necatrix* [23, 24], and *E. nieschulzi* [25]. In our previous study we demonstrated that a specific antibody to a recombinant version of EnGAM59 recognized the WFBs in macrogametes and the walls of oocysts in *Eimeria necatrix* [24]. However, it was not clear whether EnGAM59 localized to WFB1 or WFB2.

Gametocyte protein 22 (GAM22), which contains a characteristic domain rich in histidine and proline, is a second class of oocyst wall protein that has also been identified in *E. tenella* [22] and *E. necatrix* [26]. EtGAM22 was detected in mature macrogametocytes (at 168 h post-infection [p.i.]) and unsporulated oocysts of *E. tenella*, suggesting that EtGAM22, like EmGAM56, is transported to WFB2 and participates in the formation of the inner oocyst wall and/or the Stieda body [22]. A specific antibody to a recombinant version of EnGAM22 recognized the WFBs in macrogametocytes and the walls of oocysts and sporocysts of *E. necatrix* [26]. However, the exact localization of EnGAM22 remains to be evaluated.

In the present study, we used polyclonal antibodies (pAbs) raised against recombinant forms of EnGAM22 and EnGAM59 to determine the organellar location of these proteins within macrogametes and the role of these organelles in oocyst wall formation using laser confocal microscopy (LCM) and scanning electron microscopy (SEM). In addition, the extracts of WFBs and gametocytes were analyzed by sodium dodecyl sulfate–polyacrylamide gel electrophoresis (SDS-PAGE) and immunoblotting. This information will offer insights into the mechanisms governing oocyst wall formation in *E. necatrix*.

## Methods

### Parasites and animals

The Yangzhou strain of *E. necatrix* used in this study was originally isolated from a chicken that died from *E. necatrix* infection in 2009 in Yangzhou, China, as confirmed by microscopic examination and sequence analysis of the internal transcribed spacer region of genomic DNA [26]. This strain has been maintained in our laboratory. The oocysts were periodically propagated in 3–4-week-old chickens. Oocysts were isolated and harvested from the feces by salt flotation and centrifugation, sporulated in vitro at 28 °C, and stored in 2.5% potassium dichromate solution at 4 °C [26].

One-day-old chickens (purchased from the Poultry Institute of the Chinese Academy of Agricultural Sciences, Yangzhou, Jiangsu, China) were housed in *Eimeria*-free isolation cages and were provided with complete feed and clean water without anticoccidial drugs. Chicken feces were observed by salt flotation and light microscopy to ensure the absence of *Eimeria* infection prior to experimental inoculation.

Ten 4-month-old female New Zealand White rabbits (3.5–4 kg) were purchased from the Animal Genetic Engineering Laboratory at Yangzhou University. All rabbits were placed in separate cages and fed under pathogen-free conditions. The animals were allowed access to a standard rabbit diet and water ad libitum in a temperature-controlled room with a 12 h light–dark cycle at 21–23 °C and 50–75% relative humidity in the Animal Center of the College of Veterinary Medicine, Yangzhou University.

Six-week-old specific-pathogen-free female BALB/c mice were purchased from Yangzhou University (Comparative Medicine Center) and maintained under specific-pathogen-free conditions.

All animal care and procedures were conducted according to the guidelines for animal use in toxicology. The study protocol was approved by the Animal Care and Use Committee of the College of Veterinary Medicine, Yangzhou University.

### Generation of polyclonal antibodies

The recombinant proteins rEnGAM22 and rEnGAM59 were prepared using a previously published method [24, 26]. WFB protein was prepared using the method described below. Mouse anti-rEnGAM59 and WFB pAbs were prepared as described previously [24]. Briefly, 50 μg of the proteins was resuspended in 50 μl phosphate-buffered saline (PBS) and mixed with 50 μl Quick Antibody-Mouse 3W (Biodragon, Beijing, China), and then used to immunize 6-week-old BALB/c mice twice following the manufacturer’s recommendations. Blood was collected 7 days after the second immunization, centrifuged at 1500×*g* for 15 min to isolate the mouse pAbs, and stored at −80 °C.

Rabbit anti-rEnGAM22 and rEnGAM59 pAbs were generated as follows: 50 μg of the proteins was resuspended in 100 μl PBS and mixed with 100 μl Quick Antibody-Rabbit 8W (Biodragon), and then used to immunize 4-month-old New Zealand rabbits three times following the manufacturer’s recommendations. Blood was then collected 10 days after the third immunization, and the rabbit pAb was separated as described previously and stored at −80 °C.

Antibody levels were determined using an enzyme-linked immunosorbent assay (ELISA) method as described in a previous study [27]. The results showed that the optical density (OD) values of mouse anti-rEnGAM59 and anti-WFB pAbs were 3.06 (1:200 dilution) and 3.13 (1:200 dilution), whereas the OD values of rabbit anti-rEnGAM22 and anti-rEnGAM59 pAbs were 2.86 (1:200 dilution) and 2.55 (1:200 dilution), respectively. As a negative control, the OD values of naïve mice sera were less than 0.13, and the OD values of rabbit sera were less than 0.17 (Additional file 5: Table S1).

### Preparation of gametocytes

Gametocytes were isolated using previously described methods [28]. Briefly, second-generation merozoites (MZ-2) were obtained from the small intestine of chickens 136 h after oral inoculation with 2.0 × 10^4^ *E. necatrix* oocysts, and approximately 1.8 × 10^8^ MZ-2 in a volume of 2 ml was injected into the ceca of chickens, as described by McDonald and Rose [29]. At 30 ± 0.5 h after injection with MZ-2, the chickens were sacrificed and the ceca were removed and washed with cold SAC (1 mM phenylmethanesulfonyl fluoride, 1 mg/ml bovine serum albumin [BSA], 170 mM NaCl, 10 mM Tris–HCl pH 7.0, 10 mM glucose and 5 mM CaCl_2_). Then, the ceca were slit open and the mucosal tissues were scraped and incubated at 37 °C in a beaker for 2 h with 0.5 mg/ml of hyaluronidase in SAC. The digested mucosal tissues were filtered through 100, 20, and 17 μm polyester monofilament (polymon) mesh. The filtrate was centrifuged at 3000×*g* for 5 min, and the pellet was resuspended in five volumes of cold erythrocyte lysis buffer (Solarbio, Beijing, China) at 4 °C for 20 min and washed three times with cold PBS by centrifugation. The gametocytes were purified using Percoll (GE Healthcare, Uppsala, Sweden) density gradient centrifugation. Finally, the purified gametocytes were counted in a counting chamber, then immediately frozen in liquid nitrogen for future use (Additional file 1: Fig. S1). The average yield obtained was approximately 10^6^ gametocytes per infected chicken.

### Isolation and purification of WFBs

The WFBs of *E. necatrix* from macrogametocytes were purified as described previously [13], with a minor modification. Briefly, the purified *E. necatrix* gametocytes (1 × 10^8^ cells) were extracted with 0.1% saponin in Tris-NaCl-EDTA (TNE) buffer (10 mM Tris–HCl, pH 7.4; 50 mM NaCl; 2 mM ethylenediaminetetraacetic acid [EDTA]) for 20 min at room temperature and centrifuged at 1000×*g* for 5 min. The resulting pellet was washed three times in TNE by centrifugation at 3000×*g* for 2 min, and the supernatant was discarded. The pellet was resuspended in five volumes of TNE and sonicated with an output of 3.0 and duty cycle 30% for 3 s intervals over 4 min in an ice water bath. After filtering the lysates through a 5 μm polymon mesh, the filtrate was added to 5% SDS (w/v; volume ratio of filtrate to 5% SDS: 4:1), vortexed, and centrifuged at 15,000×*g* for 10 min. The WFBs in the pellet were resuspended in 5 ml TNE buffer, concentrated, and purified using a 1000 kDa cutoff Vivaspin 6 centrifugal filter (Sartorius Stedim Biotech, Aubagne, France), centrifuged at 4000×*g* for 20 min and concentrated three times. Then, the concentrated solution was collected, and the purified WFBs were obtained by centrifugation at 15,000×*g* at 4 °C for 10 min and stored at 4 °C or frozen immediately in liquid nitrogen for future use.

### Identification of WFBs

In order to confirm the identity of WFBs, the purified granules were analyzed by SDS-PAGE electrophoresis and western blot. The method is described as follows. The purified gametocytes and WFBs were resuspended in 500 μl Pierce RIPA buffer (Thermo Fisher Scientific, Waltham, MA, USA), sonicated with an output of 3.0 and duty cycle 30% in intervals of 3 s over 4 min in an ice water bath and centrifuged at 12,000×*g* at 4 °C for 10 min. Supernatants were separated, and protein concentrations of the lysates were determined using a TaKaRa Bradford Protein Assay Kit (TakaRa, Tokyo, Japan). Total protein lysates (10 μg per lane) were loaded and electrophoresed on a 12% SDS-PAGE gel. The proteins were then visualized by staining with Coomassie brilliant blue R (Sigma-Aldrich, St. Louis, MO, USA) or transferred to nitrocellulose membranes (Merck Millipore, Billerica, MA, USA) for 1.5 h at 100 V. After blocking with 3% BSA in tris-buffered saline (TBS) for 12 h at 4 °C, the membranes were incubated with mouse anti-rEnGAM59 pAb (1:400 dilution), mouse anti-WFB pAb (1:400 dilution), or rabbit anti-rEnGAM22 pAb (1:400 dilution) at room temperature for 1 h. Prior to washing three times with 0.05% Tween 20/TBS (TBST) over 30 min, the membranes were probed with peroxidase-conjugated AffiniPure goat anti-mouse immunoglobulin G (IgG) (H + L; 1:10,000; Jackson ImmunoResearch, PA, USA) or horseradish peroxidase (HRP)-labeled goat anti-rabbit IgG (H + L; 1:2500; Servicebio, Wuhan, China) and developed in the presence of High-sig ECL Western blotting substrate (Tanon, Shanghai, China) after washing with TBST. Naïve sera from mice and rabbits were used as a negative control.

### Localization of EnGAM22 and rEnGAM59 proteins in WFBs

In order to confirm the localization of EnGAM22 and rEnGAM59 proteins in WFBs of *E. necatrix*, indirect immunofluorescence assays (IFAs) were performed on purified WFBs and macrogametocytes as described previously [13, 26, 30]. Briefly, WFBs and macrogametes were placed on 0.1% (*v*/*v*) poly-l-lysine-coated glass microscope slides and fixed in methanol (− 20 °C). After blocking overnight in 5% BSA in PBS (BSA/PBS) at 4 °C, the samples were incubated with either rabbit anti-rEnGAM22 pAb (1:100 dilution) and mouse anti-rEnGAM59 pAb (1:100 dilution) or with rabbit anti-rEnGAM22 pAb (1:100 dilution) and mouse anti-WFB pAb (1:100 dilution) for 1 h at 37 °C, washed with 0.03% Tween 20/PBS (PBST) three times for 15 min and incubated with fluorescein isothiocyanate (FITC)-conjugated goat anti-rabbit IgG (1:100 dilution; MultiSciences Hangzhou, China) and Cy3-conjugated goat anti-mouse IgG (1:100 dilution; Servicebio) in BSA/PBS for 1 h at 37 °C, respectively. Before visualization, the samples were rinsed in PBST as described above. Images were obtained using LCM (Leica TCS SP8 STED [STimulated Emission Depletion], Wetzlar, Hessen, Germany). Naïve sera from rabbits and mice were used as a negative control.

### Microscopic examination of oocyst wall formation using LCM

Pathological tissue samples from chickens sacrificed 156 h p.i. were collected and processed as described in our previous study [26]. Briefly, chickens were orally infected with 30,000 *E. necatrix* sporulated oocysts and sacrificed by CO_2_ inhalation and cervical dislocation. The ceca were removed and fixed in 3% paraformaldehyde in PBS. Fixed tissues were embedded in paraffin and then cut into 5-μm-thick sections using a microtome at room temperature. The paraffin was removed from the sections prior to the inactivation of endogenous enzymes with 3% H_2_O_2_ and antigen retrieval using 0.1% trypsin (Promega Corporation, Madison WI, USA). After blocking overnight in 5% BSA in PBS (BSA/PBS) at 4 °C in a humidified chamber, the sections were incubated with either rabbit anti-rEnGAM22 pAb (1:100 dilution) or mouse anti-rEnGAM59 pAb (1:100 dilution) as described above, and then were incubated with fluorescein isothiocyanate (FITC)-conjugated goat anti-mouse antibody (dilution, 1:100; KPL) in BSA/PBS. Before visualization, the tissue sections were counterstained with 4′6-diamidino-2-phenylindole (DAPI, Beyotime, Shanghai, China). Images were obtained using LCM (Leica TCS SP8 STED, Wetzlar, Germany). The three-dimensional (3D) structure of the macrogametocytes was reconstructed from corresponding confocal images using Imaris software (Bitplane Scientific, Zurich, Switzerland). Naïve sera from mice and rabbits were used as a negative control.

### Ultrastructural examination of oocyst wall formation using SEM

The purified gametocytes were fixed in 2.5% glutaraldehyde (Sigma-Aldrich) in 0.1 M sodium cacodylate buffer. The samples were post-fixed in 1% OsO4 (Sigma-Aldrich) for 1 h, then dehydrated in a series of graded ethanol solutions (30, 50, 70, 80, 90, 95, and 100%). Subsequently, the gametocytes suspended in 100% ethanol were deposited onto a 5 mm^2^ filter paper and subjected to drying with a Leica EM CPD300 Automated Critical Point Dryer (Leica Microsystems GmbH). The dried filter paper containing the parasite samples was then meticulously placed on double-stick conducting carbon tape over an aluminum stub and sputter-coated with gold using a Leica EM SCD500 sputter coater (Leica Microsystems GmbH). The samples were examined using a ZEISS GeminiSEM 300 instrument (Carl Zeiss AG, Oberkochen, Germany).

## Results

### Protein analysis of WFBs and gametocytes

**Fig. 1:**
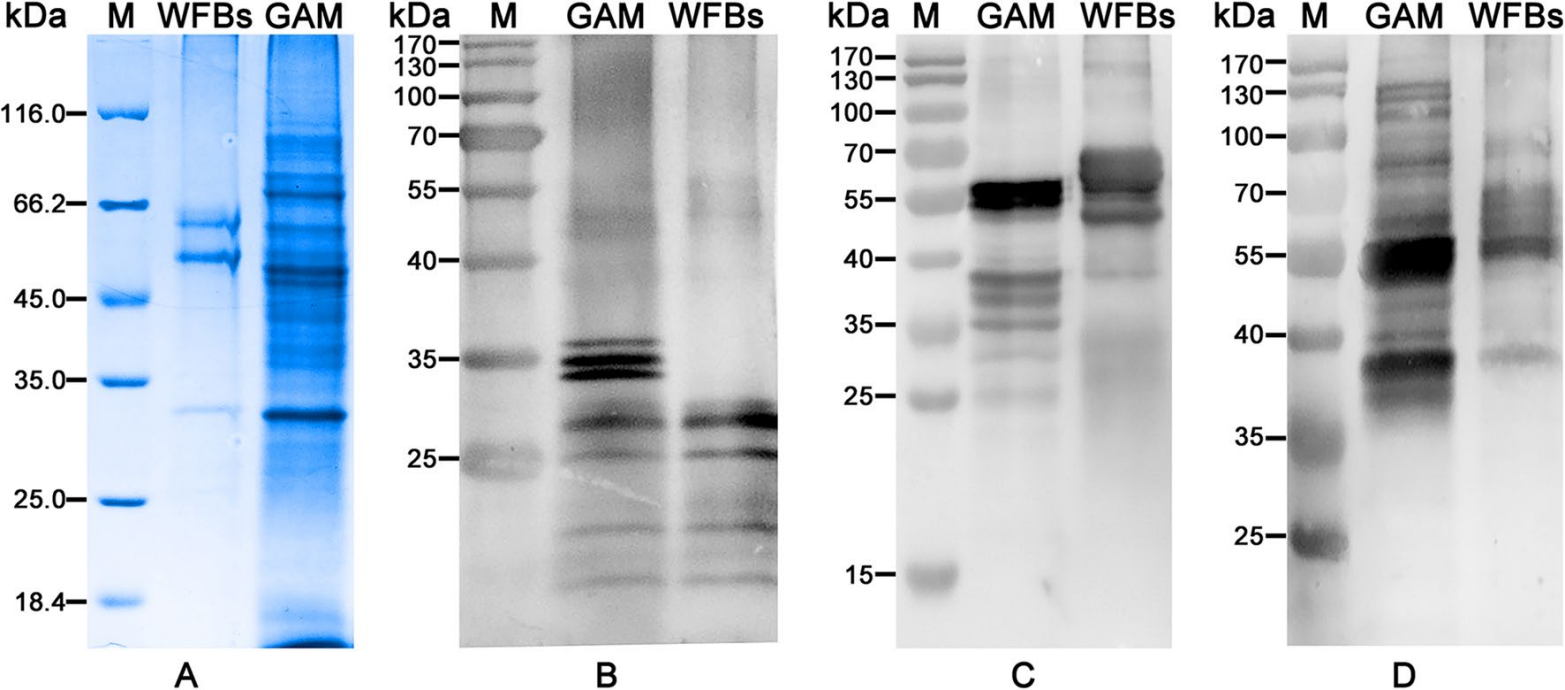
Protein analysis of WFBs by SDS-PAGE (**A**) and western blot (**B**–**D**). **A** SDS-PAGE analysis of WFBs and gametocytes (GAM). **B** Western blotting analysis with rabbit anti-rEnGAM22 polyclonal antibody. **C** Western blotting analysis with mouse anti-rEnGAM59 polyclonal antibody. **D** Western blotting analysis with mouse anti-WFB polyclonal antibody. *M* Protein marker (Lane M), *GAM* gametocyte protein (Lane GAM), *WFBs* wall-forming body proteins (Lane WFBs)

The extracts of isolated granules and gametocytes were analyzed by SDS-PAGE. Coomassie brilliant blue staining of proteins extracted from the gametocytes showed a number of protein bands migrating between 110 and 18 kDa, with the bands at 75, 68, 62, 59, 50, 48, and 33 kDa being the most prominently stained (Fig. 1A, GAM). However, only three prominent bands of 62, 59, and 33 kDa were detected in the extract prepared from WFBs (Fig. 1A, WFBs).

Western blot analysis of WFB and gametocyte extracts showed that the protein bands detected by anti-rEnGAM22 and anti-rEnGAM59 pAbs were very different (Fig. 1B, C). Using anti-rEnGAM22 pAb, four prominent bands of 36, 35, 34, and 29 kDa, along with three weak bands at 25, 22, and 21 kDa, were detected in the gametocyte lysate (Fig. 1B, GAM). However, only four bands of 29, 25, 22, and 21 kDa were detected in the WFB extract (Fig. 1B, WFB). Using anti-rEnGAM59 pAb, a prominent 55/59 protein band, along with five weak bands at 39, 37, 35, 33, and 25 kDa, were detected in the gametocyte lysate (Fig. 1C, GAM), whereas two prominent protein bands of 59/70 and 52 kDa, along with a weak band at 39 kDa, were detected in the WFB extract (Fig. 1C, WFB). In our previous studies, bands at 36, 59, 35, and 33 kDa were also detected in gametocyte lysates using anti-rEnGAM22 and anti-rEnGAM59 pAbs [23, 25]. The 36 kDa protein represents the native protein of the En*gam22* gene. The 59 kDa protein represents the native protein of the En*gam59* gene, whereas the 35 and 33 kDa proteins may represent a proteolytically processed product of EnGAM59.

Western blot analysis also revealed that both anti-WFB and anti-rEnGAM59 pAbs recognized two protein bands of 59 and 39 kDa from the WFB and gametocyte extracts. However, more protein bands were detected from gametocyte lysates by anti-WFB pAb (Fig. 1C, D). In addition, two bands of 52/59 and 39 kDa, multiple weak bands migrating between 130 and 62 kDa, and three weak bands at 45, 40, and 38 kDa were detected in the gametocyte lysate by the anti-WFB pAb (Fig. 1D, GAM).

As a control, the WFB and gametocyte extracts probed with negative control sera showed no reactivity (Additional file 2: Fig. S2A, B). These results confirmed that the granules isolated from gametocytes were WFBs of *E. necatrix*.

### Localization of EnGAM22 and EnGAM59

**Fig. 2:**
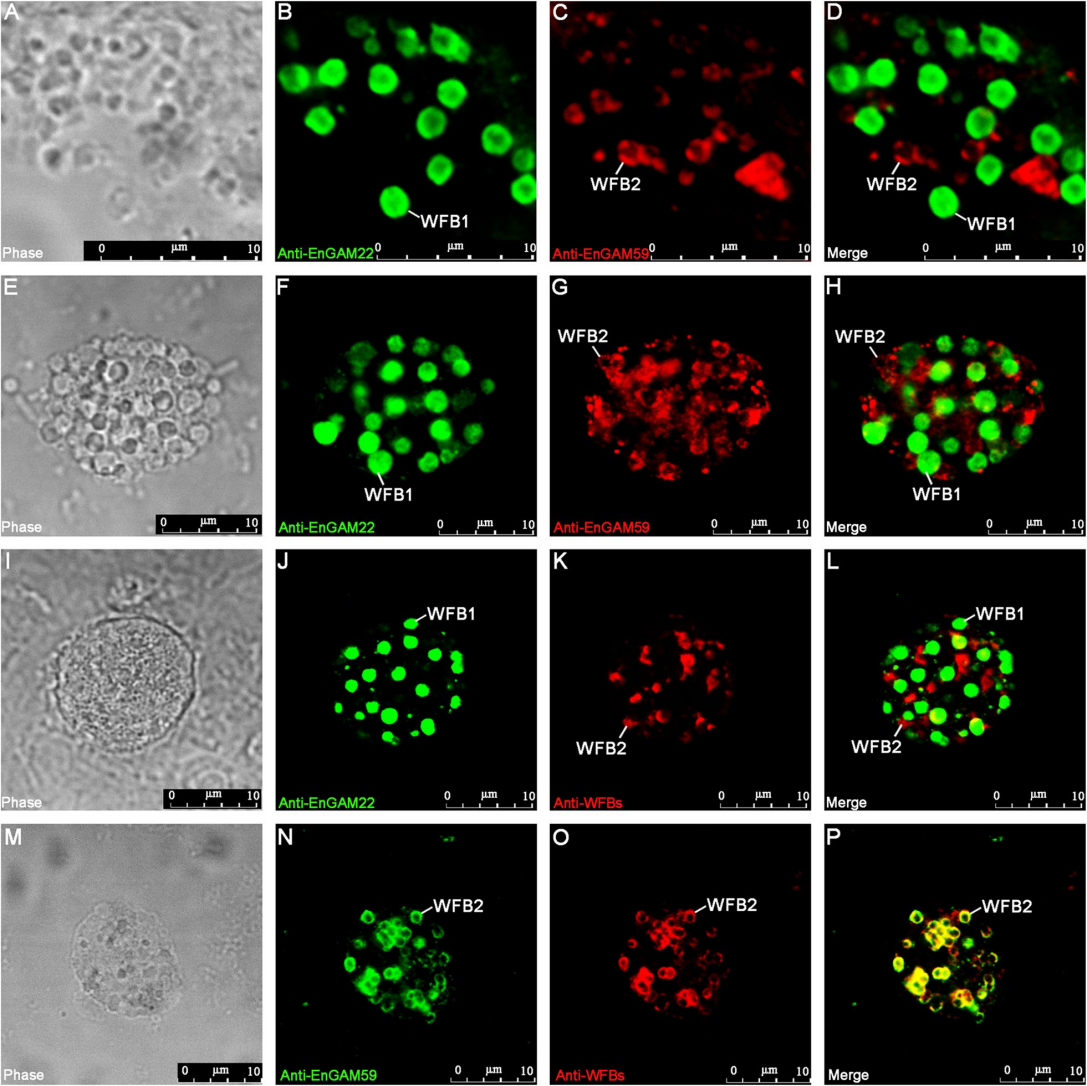
Laser confocal microscopy (LCM) of WFBs and macrogametocytes. **A**–**D** Immunofluorescence co-staining of EnGAM22 and EnGAM59 in WFBs. **A** Bright-field images of the WFB-rich extract. **B** Fluorescent micrographs of the WFBs incubated with rabbit anti-rEnGAM22 pAb (visualized with FITC, green). **C** Fluorescent micrographs of the WFBs incubated with mouse anti-rEnGAM59 pAb (visualized with Cy3, red). **D** Merged image of (**B**) and (**C**). This shows that the enriched fractions include both intact WFB1s and WFB2s. **E**–**H** Immunofluorescence co-staining of EnGAM22 and EnGAM59 in macrogametocytes. **E** Bright-field images of the macrogametocytes. **F** Fluorescent micrographs of the macrogametocytes incubated with rabbit anti-rEnGAM22 pAb (visualized with FITC, green). **G** Fluorescent micrographs of the macrogametocytes incubated with mouse anti-rEnGAM59 pAb (visualized with Cy3, red). **H** Merged image of (**F**) and (**G**). **I**–**L** Immunofluorescence co-staining of EnGAM22 and WFB proteins in macrogametocytes. **I** Bright-field images of the macrogametocytes. **J** Fluorescent micrographs of the macrogametocytes incubated with rabbit anti-rEnGAM22 pAb (visualized with FITC, green). **K** Fluorescent micrographs of the macrogametocytes incubated with mouse anti-WFB pAb (visualized with Cy3, red). **L** Merged image of (**J**) and (**K**). **M**–**P** Immunofluorescence co-staining of EnGAM59 and WFB proteins in macrogametocytes. **M** Bright-field images of the macrogametocytes. **N** Fluorescent micrographs of the macrogametocytes incubated with rabbit anti-rEnGAM59 pAb (visualized with FITC, green). (**O**) Fluorescent micrographs of the macrogametocytes incubated with mouse anti-WFB pAb (visualized with Cy3, red). **P** Merged image of (**N**) and (**O**). Scale bars represent 10 μm

The resulting pAbs were tested on the purified granules and gametocytes of *E. necatrix* by IFA to study the subcellular localization of the EnGAM22 and EnGAM59 proteins. The purified granules reacted with rabbit anti-EnGAM22 pAb and mouse anti-EnGAM59 pAb, respectively (Fig. 2A−D). Anti-EnGAM22 pAb reacted exclusively with spherical granules with dense contents and an average size of 1790 × 1642 nm (range, 806–2360 × 659–2187 nm, *n* = 50) (Fig. 2B), whereas anti-EnGAM59 pAb reacted exclusively with “doughnut-shaped” granules with an average size of 1110 × 928 nm (range, 410–1889 × 344–1507 nm, *n* = 50) (Fig. 2C). The same results were obtained from the isolated macrogametocytes (Fig. 2E−H). The spherical granules with dense contents or with a doughnut-shaped appearance were predicted to be WFB1 or WFB2, respectively, as described in previous studies [12, 13]. Therefore, these results further confirmed that the granules isolated from gametocytes were the WFBs and EnGAM22 and EnGAM59 proteins localized to the WFB1 and WFB2 of *E. necatrix*. Interestingly, anti-WFB pAb reacted only with WFB2 (Fig. 2I−L and M−P), which was consistent with the result that anti-WFB pAb did not recognize the EnGAM22 protein from the extracts of WFBs and macrogametocytes (Fig. 1D).

### Macrogametocyte development and oocyst wall formation

**Fig. 3:**
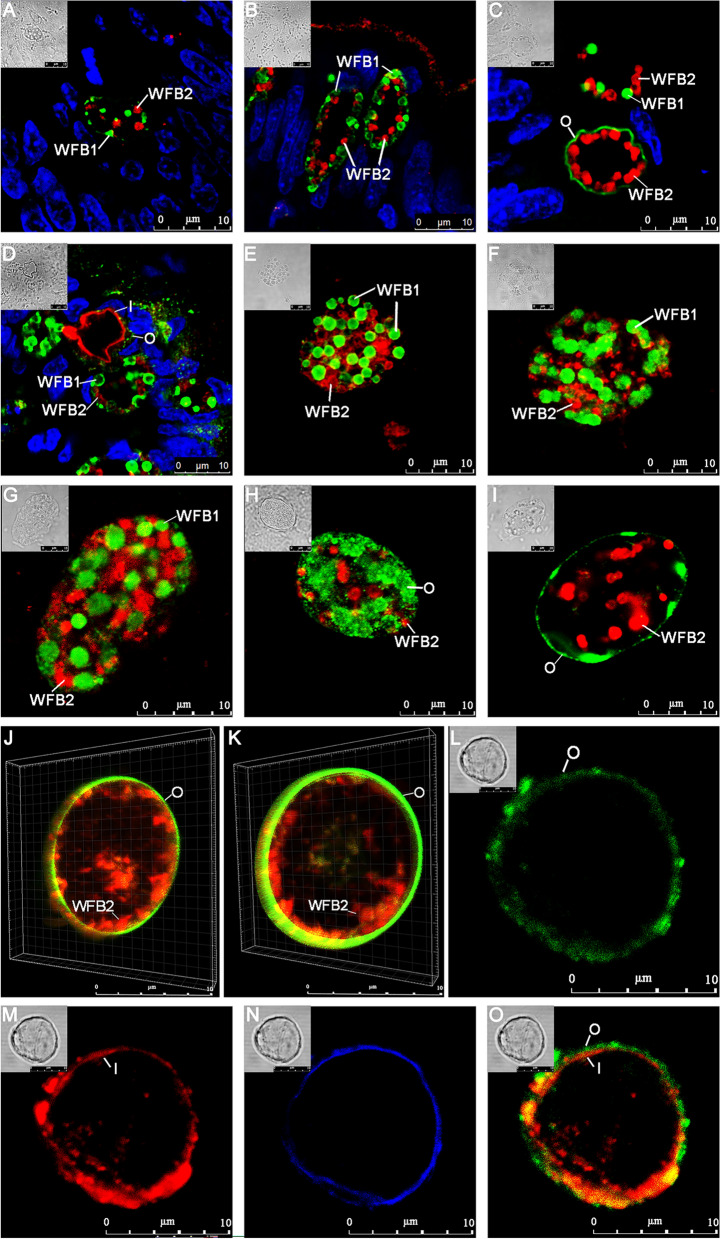
Laser confocal microscopy (LCM) of macrogametes in situ (**A**–**D**) and harvested freshly (**E**–**K**) and fully formed oocyst (**L**–**O**) of *E. necatrix* (immuno-labeled with rabbit anti-rEnGAM22 pAb [visualized with FITC, green] and mouse anti-rEnGAM59 pAb [visualized with Cy3, red]). **A**–**D** Macrogametocyte genesis of *Eimeria necatrix* in tissue sections of chicken intestine 156 h p.i., counterstained with DAPI. **A** An early-stage in situ macrogametocyte showing several WFB1s (green) and WFB2s (red) dispersed in the cytoplasm. **B** A mature macrogametocyte in situ showing WFB1 membrane-bound in the peripheral cytoplasm and numbers of WFB2s underneath the WFB1s. **C** Late-stage in situ macrogametocytes showing that WFB1 formed in the outer oocyst wall, and WFB2s were underneath the outer oocyst wall and linked together. **D** An early oocyst in situ showing WFB2s formed in the inner wall. **E** A mid-stage harvested macrogametocyte exhibits spheroidal-shaped WFBs distributed throughout the cytoplasm. **F**, **G** The isolated mature macrogametocyte showing that WFB1s were arranged in necklace-like structures, while the WFB2s were dispersed throughout the cytoplasm. **H**, **I** The late-stage harvested macrogametocyte showing that WFB1s align at the parasite periphery and exocytose their contents to form the outer oocyst wall, while WFB2s lose their substructure and fuse into amorphous material that coalesces into small islands. **J**, **K** 3D confocal microscopy images of the forming oocyst revealed that WFB2s were located beneath the outer oocyst wall and fused to form an amorphous material. (**L**–**O**) The fully formed oocyst immunofluorescence co-localization of rabbit anti-rEnGAM22 [image (**L**)] and mouse anti-rEnGAM59 [image (**M**)]. **N**, UV autofluorescence (blue) of oocyst wall. Image (**O**) represents the merged image of (**L**) and (**M**), showing green fluorescence layer covering the surface of the red fluorescence layer. *WFB1* wall-forming body 1, *WFB2* wall-forming body 2, *O* outer oocyst wall, *I* inner oocyst wall. Bar represents 10 μm

To gain further insights into macrogametocyte development and oocyst wall formation in *E. necatrix*, macrogametocytes in tissue samples (Fig. 3A–D) or isolated from tissue (Fig. 3E–K) and unsporulated oocysts (Fig. 3L–O) were examined by IFA with rabbit anti-rEnGAM22 pAb and mouse anti-rEnGAM59 pAb. Early macrogametocytes appeared to contain several WFB1s (green) and WFB2s (red) dispersed in the cytoplasm (Fig. 3A). In slightly later-stage or mid-stage macrogametocytes, the number and size of WFBs increased, and the staining intensity of WFB1 was greater than that seen for WFB2, which appeared as characteristic doughnut-shaped granules (Fig. 3E). In mature macrogametocytes, WFB1s were located around the periphery of the parasite (Fig. 3B) or arranged in “necklace-like” structures in the peripheral cytoplasm (Fig. 3F), whereas WFB2s appeared around WFB1s (Fig. 3B) or dispersed in the cytoplasm (Fig. 3G).

With the further development of macrogametocytes to oocysts, WFB1s located in the peripheral cytoplasm aligned and fused together to form amorphous patches (Fig. 3G, H). Subsequently, the amorphous patches linked together to form the outer oocyst wall. At the same time, WFB2s were situated directly underneath the outer oocyst layer and aggregated into “necklace-like” structures (Fig. 3C). WFB2s lost their doughnut-shaped substructure and fused together to form an amorphous material (Fig. 3G, H) that then coalesced into small islands of amorphous material (Fig. 3I). The gametocytes were then analyzed by 3D LCM to observe the cell geometry and three-dimensional shape of the WFB2s at this process (Fig. 3J, K, Additional file 3: Movie S1, Additional file 4: Movie S2). The results revealed that these islands became further cross-linked to form large, fused WFBs, finally becoming the inner oocyst wall (Fig. 3D). In fully formed oocysts, the outer layer of the oocyst wall appeared to react positively with rabbit anti-rEnGAM22 pAb (Fig. 3L), whereas the inner layer of the oocyst wall appeared to react positively with mouse anti-rEnGAM59 pAb (Fig. 3M). When examined microscopically using an ultraviolet (UV) excitation wavelength of 330–385 nm, the oocysts showed blue autofluorescence, which represented the inner layer of the oocyst wall (Fig. 3N). When the two images (Fig. 3L, M) were merged, the merged image revealed that the green fluorescence layer was located directly on the surface of the red fluorescence layer (Fig. 3O), further confirming that EnGAM22 and EnGAM59 proteins participated in the formation of the outer and inner layers of the oocyst wall, respectively.

**Fig. 4:**
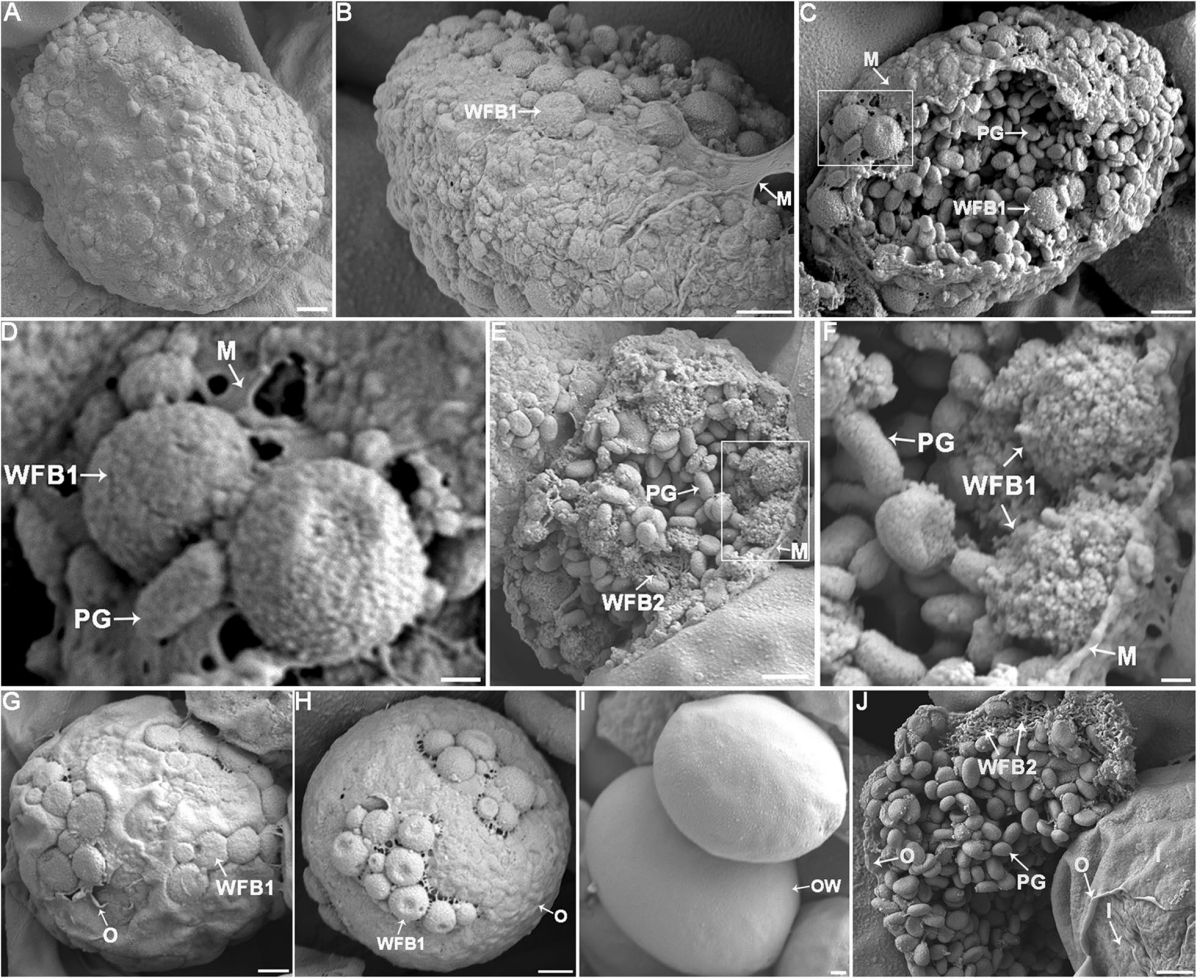
Scanning electron microscopy (SEM) images of macrogametocytes and oocyst of *E. necatrix*. **A** The complete macrogametocyte is enveloped by a thin layer of plasma membrane on its surface. **B** A macrogametocyte with a ruptured plasma membrane, with spherical granules, predicted as WFB1s, scattered beneath the membrane. **C** A ruptured plasma membrane of a macrogametocyte revealed a mulberry-like appearance, exposing numerous WFB1s and PGs on the surface, and the PGs also filled the cytoplasm. **D** Enlargement of the section displayed in (**C**) showing WFB1s appearing as spherical dense granules with a relatively smooth surface and approximately 1 μm diameter. **E** A macrogametocyte with a ruptured plasma membrane, exposing its internal structure. Numerous WFB1s were observed arranged beneath the plasma membrane, with PGs distributed throughout the cytoplasm. Additionally, sponge-like structures were observed inside sectioned organelles within the cytoplasm of the parasite, which were predicted as WFB2. **F** Enlargement of the section displayed in (**E**) showing WFB1s 0.8−1.0 μm in diameter. **G** An intact macrogametocyte, with WFB1s horizontally flattened and fused together on the surface. Gaps and suture structures were observed in the irregular, incompletely formed outer layer. **H** An intact macrogametocyte, where the outer layer of the oocyst wall has partially formed, and the remaining WFB1s are interconnected on the surface. **I** An intact oocyst, where the WFB1s finally synthesized the smooth and flat outer oocyst wall. **J** At the center of the image, a macrogametocyte with a ruptured outer oocyst wall displays cross-linked structures formed by WFB2s. In the lower right corner of the image, an oocyst with a partially stripped outer oocyst wall exposes the inner oocyst wall that has already formed. *PG* polysaccharide granule, *WFB1* wall-forming body 1, *WFB2* wall-forming body 2, *M* membrane, *O* outer oocyst wall, *I* inner oocyst wall, *OW* oocyst wall. Scale bars represent (**A**, **B**, **C**, **E**, **G**, **H**, **I**, **J**) 1 μm, (**D**, **F**) 200 nm

To better understand the steric structure and microscopic morphology of macrogametes during oocyst wall formation in *E. necatrix*, the macrogametocytes were purified and observed using SEM. The SEM images were interpreted with the observations obtained using LCM above and transmission electron microscopy (TEM) data from previously published studies [3, 5–11, 19, 21, 32–34]. As shown in Fig. 4, the mature macrogametocytes had a “mulberry-like” appearance, with a number of granules on the surface (Fig. 4A, B, C). Some of these particles were located directly underneath the plasma membrane of macrogametocytes (Fig. 4B, E, F) and appeared as dense spherical granules with an average size of 1035 × 923 nm (range, 588–1457 × 540–1123 nm, *n* = 50). By comparison with the images of WFB1 under TEM [3, 12, 21, 31, 32], these granules were predicted to be WFB1 of *E. necatrix*. In addition, there were a large number of flat oval granules with an average size of 631 × 430 nm (range, 426–964 × 334–558 nm, *n* = 50), most of which were located in the cytoplasm of the macrogamete (Fig. 4C, E, F, J), and a few of which were located on the macrogamete surface (Fig. 4D). Based on previous TEM findings that the polysaccharide granules (PGs) appeared as ovoid and measured approximately 500 nm by 250 nm in the macrogamete and in developing oocysts distributed throughout the cytoplasm of the macrogamete [32, 39], these flat oval granules were predicted to be PGs of *E. necatrix*. In the cytoplasm of the macrogamete (Fig. 4E) and underneath the outer layer of the oocyst wall (Fig. 4J), there were also some sponge-like or network structures formed by filaments. According to TEM literature reports [7, 8, 10, 12, 32], WFB2s start as irregular electron-dense deposits in the rough endoplasmic reticulum with a sponge-like appearance, and later become labyrinthine and appear as electron-dense bodies, with the formation of an overlapping ring internal to the WFB1 ring. Therefore, these network structures were predicted as WFB2 of *E. necatrix*. During the process of oocyst wall formation, WFB1s appeared to be horizontally flattened and fused together to form an outer layer of the oocyst wall (Fig. 4G–I). Concurrently, WFB2s may have agglomerated to form cross-linked network structures and finally formed an inner layer of the oocyst wall (Fig. 4J).

## Discussion

WFB1 and WFB2 are two specific organelles that are found exclusively in the sexual, macrogamete stage of coccidian parasites, and the contents of WFBs form the oocyst wall [32–34]. Initially, the WFB1s form as dense granules while WFB2s develop within the rough endoplasmic reticulum and are retained until after the secretion of the WFB1 [3, 12]. In chicken *Eimeria*, WFB1 appears as an electron-dense, membrane-bound structure that is larger than WFB2, whereas in the early stages, WFB2s appear as amorphous contents and finally appear as whorled or “doughnut-shaped” substructures with amorphous electron-dense contents [3, 11, 12]. In this study, the analysis of the purified granules using IFA showed that rabbit anti-EnGAM22 pAb reacted exclusively with spherical granules with dense content, and mouse anti-EnGAM59 pAb reacted exclusively with doughnut-shaped granules. This led us to conclude that the granules isolated from gametocytes contain WFBs of *E. necatrix*. These results also suggest that the structure of WFB1 and WFB2 is strongly conserved across avian *Eimeria* species.

In our previous studies, proteins from gametocyte lysates were analyzed using anti-EnGAM22 and anti-EnGAM59 [24, 26]. In the present study, we used immunoblotting to compare proteins from the isolated gametocytes with those from purified WFBs using anti-EnGAM22, anti-EnGAM59, and anti-WFB pAbs. Using anti-rEnGAM22 pAb, we found only four smaller-molecular-weight proteins in the extracted WFBs and the complete absence of larger-molecular-weight proteins, including the native protein of ~ 36 kDa. This may be explained by the proteolytic processing of EnGAM22 proteins in WFB1 in *E. necatrix*. Using anti-WFB pAb, more protein bands were detected in gametocyte lysates than in WFB extracts, but both showed the complete absence of protein bands with a molecular weight less than 39 kDa. One possible explanation for this result was that, due to their lower content or absence in WFB extracts, there were no antibodies against proteins such as EnGAM22 in the developed anti-WFB pAb. In fact, the strong anionic surfactant SDS used to purify WFBs may destroy the lipid-rich organelles of WFB1s and cause the loss of some antigens [30].

Previous studies have shown that WFB2 characteristically reacts with anti-GAM56, anti-GAM82, and anti-GAM230 pAbs, with anti-GAM56 and anti-GAM82 pAbs appearing to react more strongly with the whorled or doughnut-shaped structures that form groups within WFB2, and anti-GAM230 pAb appearing to stain the material around the cores [12, 19, 21]. EnGAM59 protein, a second GAM56-like protein similar to GAM56 and GAM82 of other chicken *Eimeria*, contains a tyrosine-rich domain [22, 24]. As expected, EnGAM59 was localized to WFB2 and the inner oocyst wall of *E. necatrix*, and WFB2 stained with anti-EnGAM59 pAb had a whorled or doughnut-shaped appearance.

The structure of WFB1 is typical of storage granules and has long been thought to contain mucoproteins, mucopolysaccharides, and glycoproteins [34, 35]. A recent study reported that WFB1 contains a lipid core with a protein-rich surface coat and that the outer oocyst wall, like WFB1, is mainly composed of neutral lipids such as triglycerides and cholesterol [13]. However, few molecules have been identified from the WFB1 of *Eimeria*. Recently, a study focusing on RNA sequencing (RNA-seq) analysis of the *E. tenella* gametocyte transcriptome showed that a cysteine-rich oocyst wall protein named EtOWP6 localized to WFB1 but not to WFB2 [36]. In the present study, anti-EnGAM22 pAb recognized WFB1 but not WFB2. Unstained WFB2 was identified by counterstaining with anti-EnGAM59 pAb. Immunolocalization studies of *E. necatrix*-infected chicken ceca (156 h p.i.) and macrogametes isolated using anti-EnGAM22 pAb revealed reactivity against WFB1 and the outer oocyst wall, with anti-EnGAM59 pAb used as a counterstain for WFB2 and the inner oocyst wall. These results suggest that EnGAM22 is transported to WFB1 and participates in the formation of the outer oocyst wall.

A previous study reported that both WFB1 and WFB2 in macrogametocytes of *E. maxima* were intensely stained by anti-WFB pAb, with WFB1 appearing as a large ring with a central luminal hole and WFB2 having a doughnut-shaped appearance [30]. In the present study, the anti-WFB pAb reacted exclusively with WFB2 in isolated macrogametocytes of *E. necatrix*. The WFB2 labeled with anti-WFB had a doughnut-shaped appearance similar to that seen with anti-EnGAM59. These results were also supported by immunoblotting with anti-WFB pAb, in which a predominant protein band of 59 kDa was identified from both WFB and gametocyte extracts, but no protein bands with a molecular weight less than 39 kDa were detected.

Immunofluorescence with anti-GAM22 and anti-GAM59 as markers revealed that macrogametocyte development and oocyst wall formation in *E. necatrix* were similar to those of *E. maxima* [12, 19], *E. tenella* [19, 20], *E. acervulina* [19], and *E. nieschulzi* [21], revealing that the structure of macrogametocytes and the formation of the oocyst wall are strongly conserved across *Eimeria* species. Further observations using SEM showed that WFB1 appeared as dense spherical granules and localized to the surface of macrogametocytes, then fused together to form an outer layer of the oocyst wall. WFB2s appeared as sponge-like or network structures formed by filaments. Following the formation of the outer oocyst wall, WFB2s aggregate to form cross-linked network structures. This finding is consistent with previous reports [7, 8, 10, 12, 32].

Previous studies have reported that GAM precursor proteins (GAM56, GAM82) containing tyrosine-rich domains are proteolytically processed into smaller peptides prior to protein-tyrosine cross-linking and oocyst wall hardening [3, 18]. Hence, dityrosine cross-linking and hardening of the oocyst wall lead to characteristic blue UV autofluorescence [3, 18, 37]. In this study, only the inner layer of the oocyst wall that reacted positively with mouse anti-rEnGAM59 exhibited blue autofluorescence. However, the participation of EnGAM59 in oocyst wall formation requires further investigation.

## Conclusion

In summary, we successfully purified WFBs from *E. necatrix* macrogametocytes and found that EnGAM22 and EnGAM59 localized to WFB1 and WFB2, respectively. EnGAM22 is transported to WFB1 and participates in the formation of the outer oocyst wall, and anti-GAM22 pAbs can be used as a tool to follow the transport and secretion of proteins in WFB1s during oocyst development. Combining LCM and SEM, we found that the processes of macrogametogenesis and oocyst wall formation of *E. necatrix* are similar to other *Eimeria* parasites and furthered our understanding of the mechanisms of oocyst wall formation. Hopefully, subsequent research on WFB antigens will enable the development of transmission-blocking immunizations.

## Supplementary Information

**Additional file 1: Fig. S1.** Purified macrogametocytes of *E. necatrix *observed by light microscope. Bar represents 20 μm.

**Additional file 2: Fig. S2.** Negative controls of western blotting analysis with normal mouse serum (**A**) and normal rabbit serum (**B**). M: Protein marker (Lane M), GAM: gametocyte protein (Lane GAM), WFBs: wall-forming body protein (Lane WFBs).

**Additional file 3: Movie S1.** The isolated late-stage macrogametocyte observed by 3D confocal microscopy. Green channel is EnGAM22, red channel is EnGAM59, bar represents 2 μm, related to Fig. 3J.

**Additional file 4: Movie S2.** The isolated late-stage macrogametocyte observed by 3D confocal microscopy. Green channel is EnGAM22, red channel is EnGAM59, bar represents 2 μm, related to Fig. 3K.

**Additional file 5: Table S1.** ELISA results of polyclonal antibody.

## Data Availability

All data and materials of the experiments described here are included in this published article and its additional files.

## Abbreviations

WFBs: Wall-forming bodies
LCM: Laser confocal microscopy
SEM: Scanning electron microscope
SDS-PAGE: Sodium dodecyl sulfate–polyacrylamide gel electrophoresis
pAb: Polyclonal antibodies
PBS: Phosphate-buffered saline
ELISA: Enzyme-linked immunosorbent assay
OD: Optical density
MZ-2: Second-generation merozoites
BSA: Bovine serum albumin
TBS: Tris-buffered saline
TEM: Transmission electron microscope
IFA: Immunofluorescence assay
PG: Polysaccharide granules
