# Supplementary figures and images for: Examination of gametocyte protein 22 localization and oocyst wall formation in Eimeria necatrix using laser confocal microscopy and scanning electron microscopy
Source: Parasit Vectors. 2023 Apr 12;16:124. doi: 10.1186/s13071-023-05742-z (PMC10091644; doi:10.1186/s13071-023-05742-z)

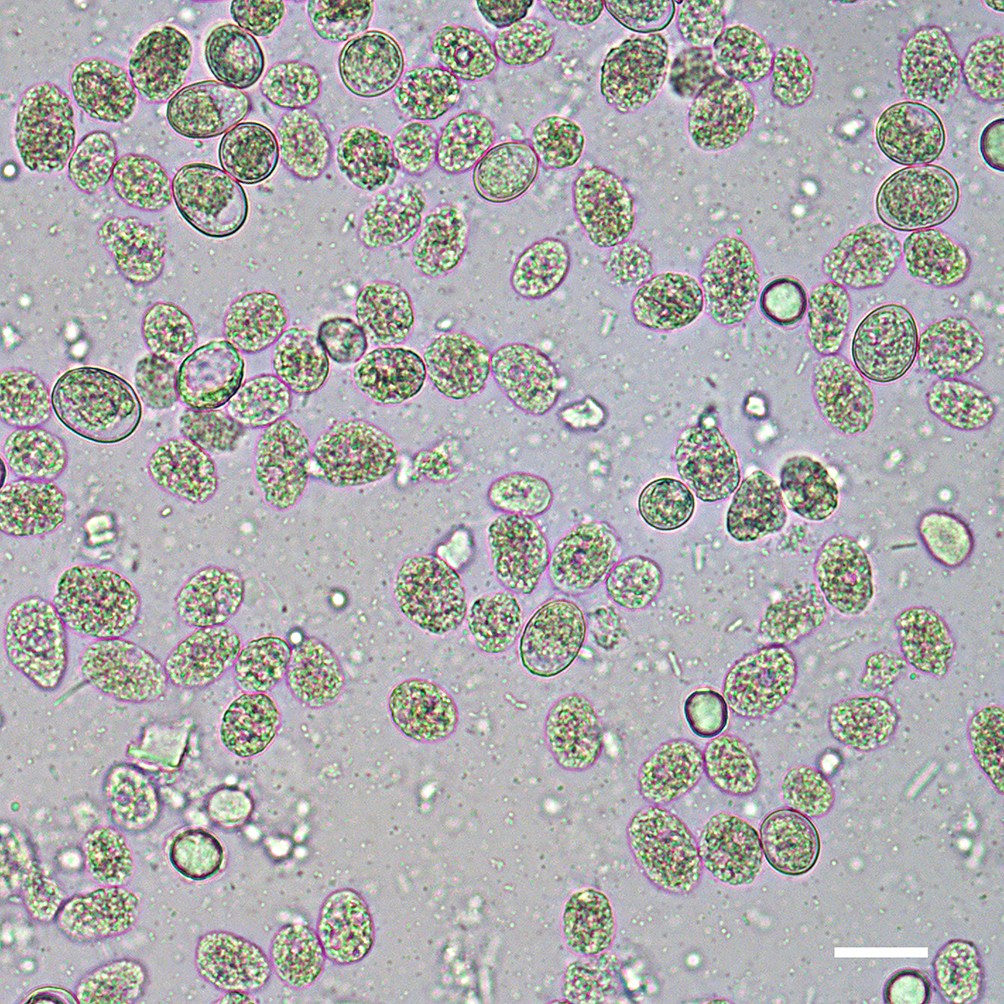

Supplement: Supplementary file 1 — Additional file 1: Fig. S1. Purified macrogametocytes of E. necatrix observed by light microscope. Bar represents 20 μm. [file 13071_2023_5742_MOESM1_ESM.tif]

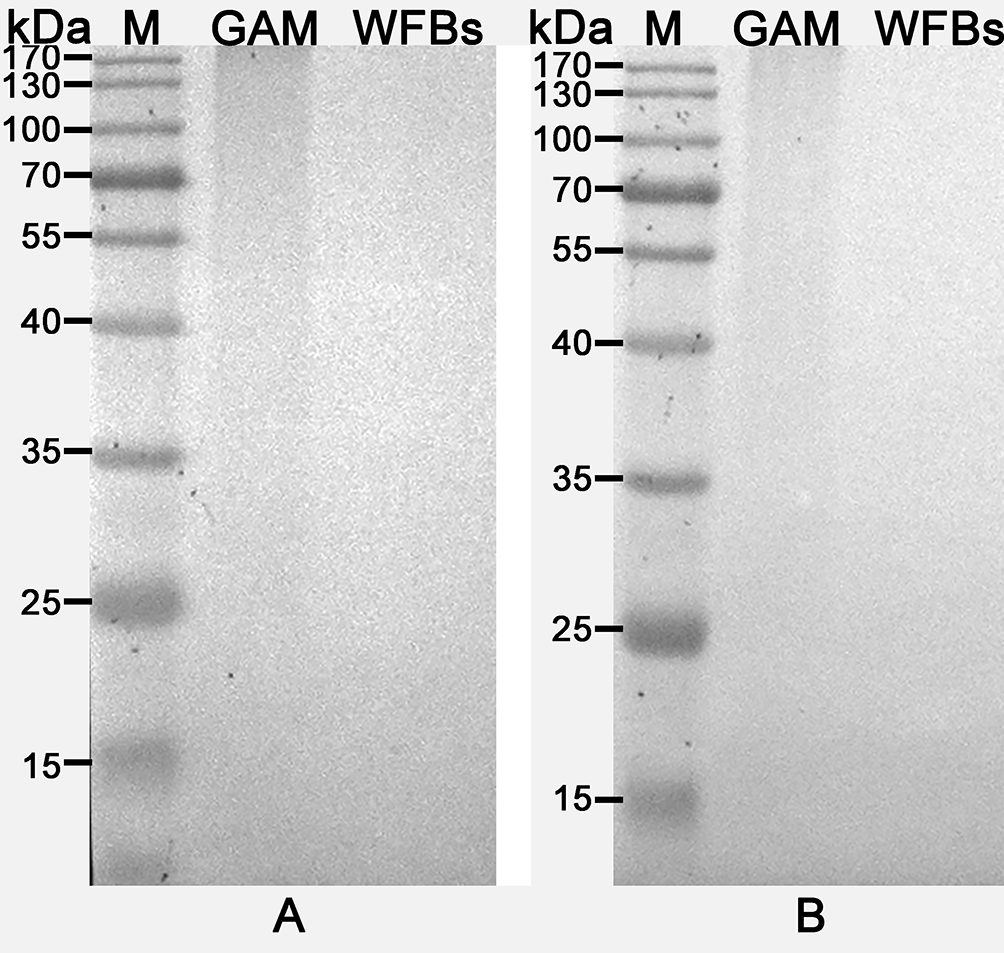

Supplement: Supplementary file 2 — Additional file 2: Fig. S2. Negative controls of western blotting analysis with normal mouse serum (A) and normal rabbit serum (B). M: Protein marker (Lane M), GAM: gametocyte protein (Lane GAM), WFBs: wall-forming body protein (Lane WFBs). [file 13071_2023_5742_MOESM2_ESM.tif]
