# Supplementary material for: Examination of gametocyte protein 22 localization and oocyst wall formation in Eimeria necatrix using laser confocal microscopy and scanning electron microscopy
Source: Parasit Vectors. 2023 Apr 12;16:124. doi: 10.1186/s13071-023-05742-z (PMC10091644; doi:10.1186/s13071-023-05742-z)
Supplement: Supplementary file 5 — Additional file 5: Table S1. ELISA results of polyclonal antibody. [file 13071_2023_5742_MOESM5_ESM.docx]

**Additional file 1: Table S1** ELISA Results of Polyclonal Antibody

| Type of antibody | OD_450_(mean) | SD(±) |
| --- | --- | --- |
| Mouse anti-EnGAM59 | 3.06 | 0.02 |
| Mouse negative serum | 0.10 | 0.01 |
| Mouse anti-WFBs | 3.13 | 0.05 |
| Mouse negative serum | 0.13 | 0.01 |
| Rabbit anti-EnGAM22 | 2.86 | 0.10 |
| Rabbit negative serum | 0.17 | 0.01 |
| Rabbit anti-EnGAM59 | 2.55 | 0.14 |
| Rabbit negative serum | 0.16 | 0.03 |

OD: optical density;

SD: standard deviation.
